# Supplementary figures and images for: The 1B vaccine strain of Chlamydia abortus produces placental pathology indistinguishable from a wild type infection
Source: PLoS One. 2020 Nov 16;15(11):e0242526. doi: 10.1371/journal.pone.0242526 (PMC7668586; doi:10.1371/journal.pone.0242526)

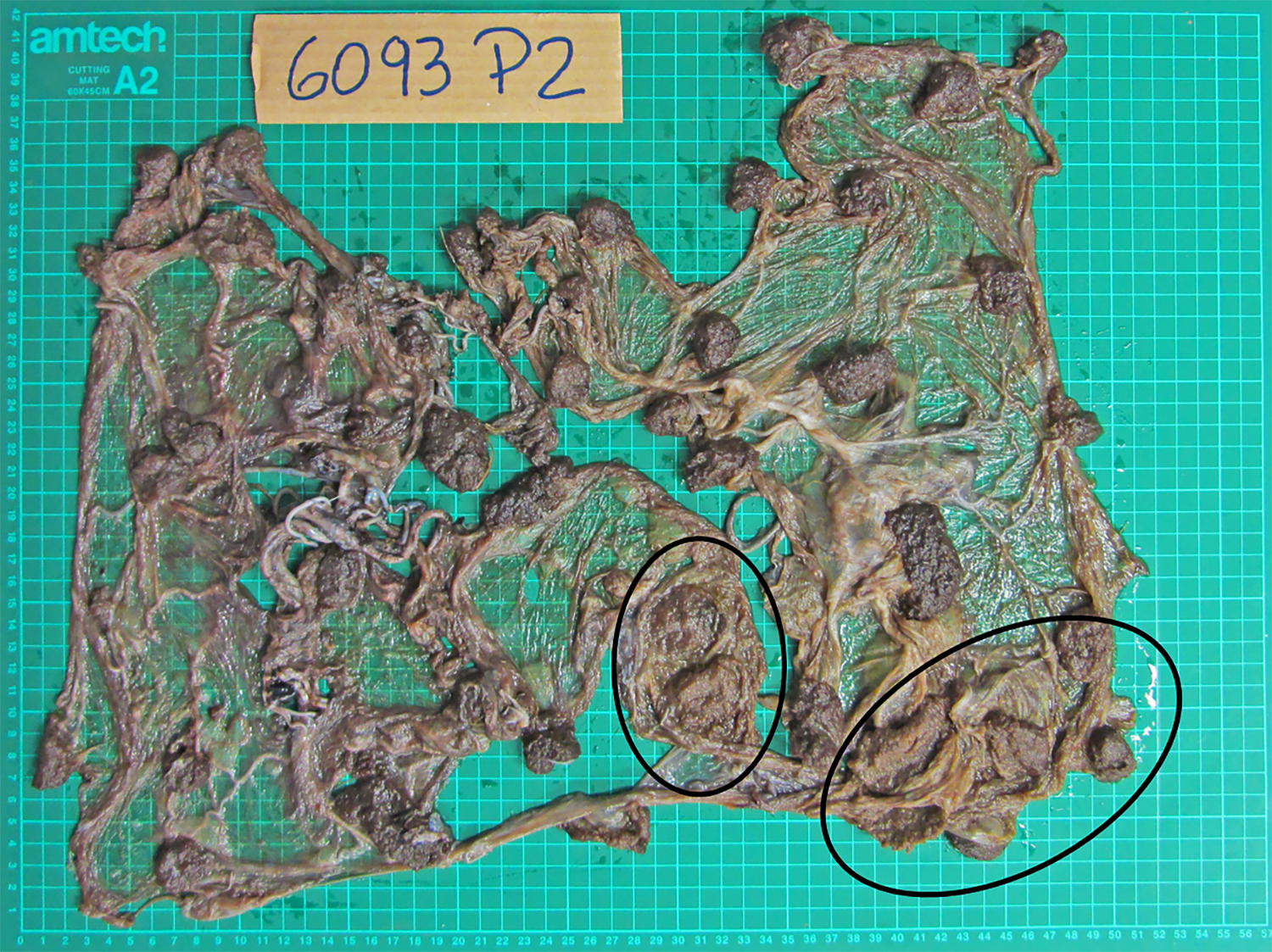

Supplement: S1 Fig — Placenta vt-P2, note the difference in the coloration of the cotyledons and the thickening of the intercotyledonary membranes of the marked areas (black circles). (TIF) [file pone.0242526.s001.tif]

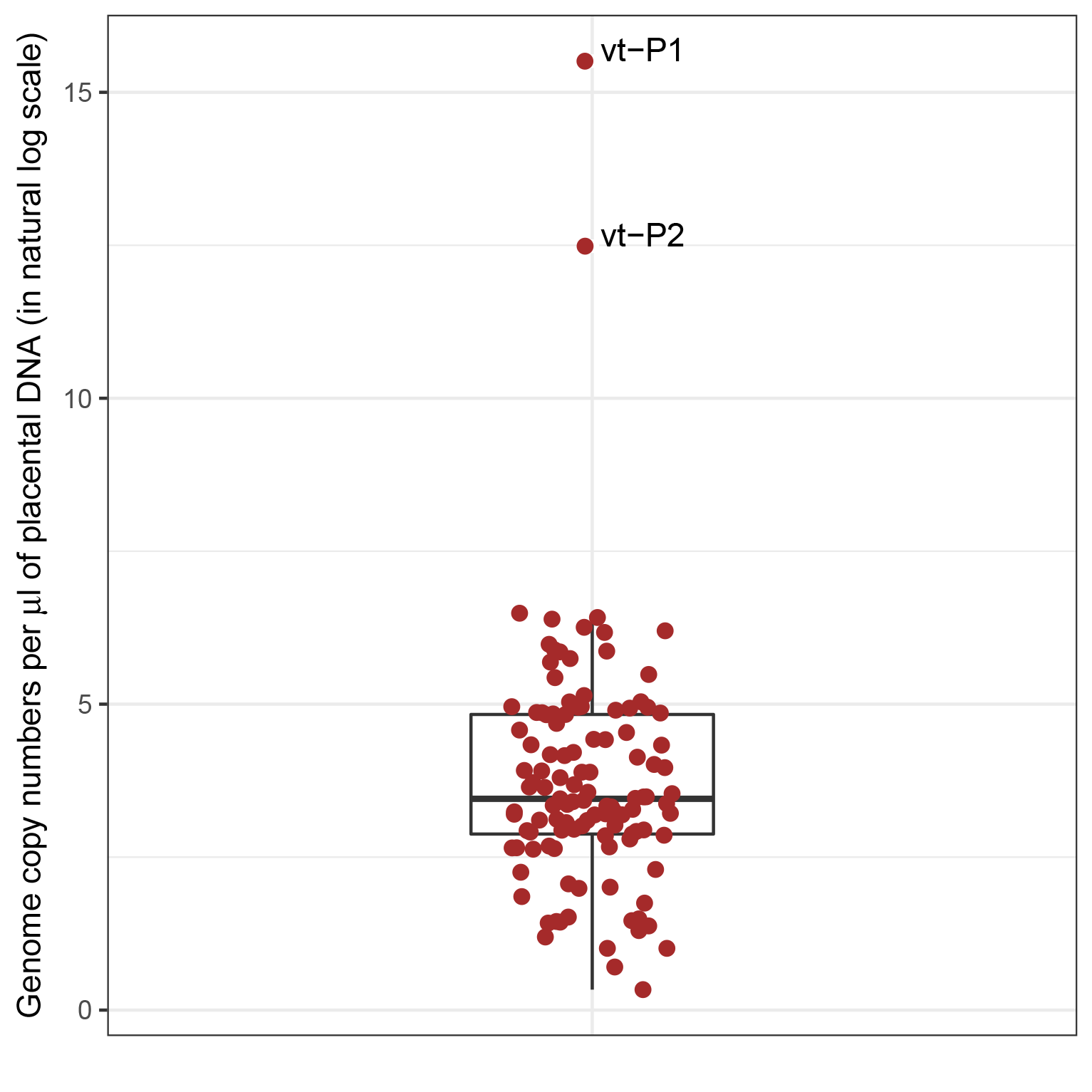

Supplement: S2 Fig — Genome copies of C. abortus per μl of placental DNA. Boxplot showing the number of genome copies (natural logarithmic scale) of C. abortus per μl of extracted genomic DNA in the 117 placentas analysed by qPCR. The two outlier points correspond to vt-P1 and vt-P2. (TIF) [file pone.0242526.s002.tif]

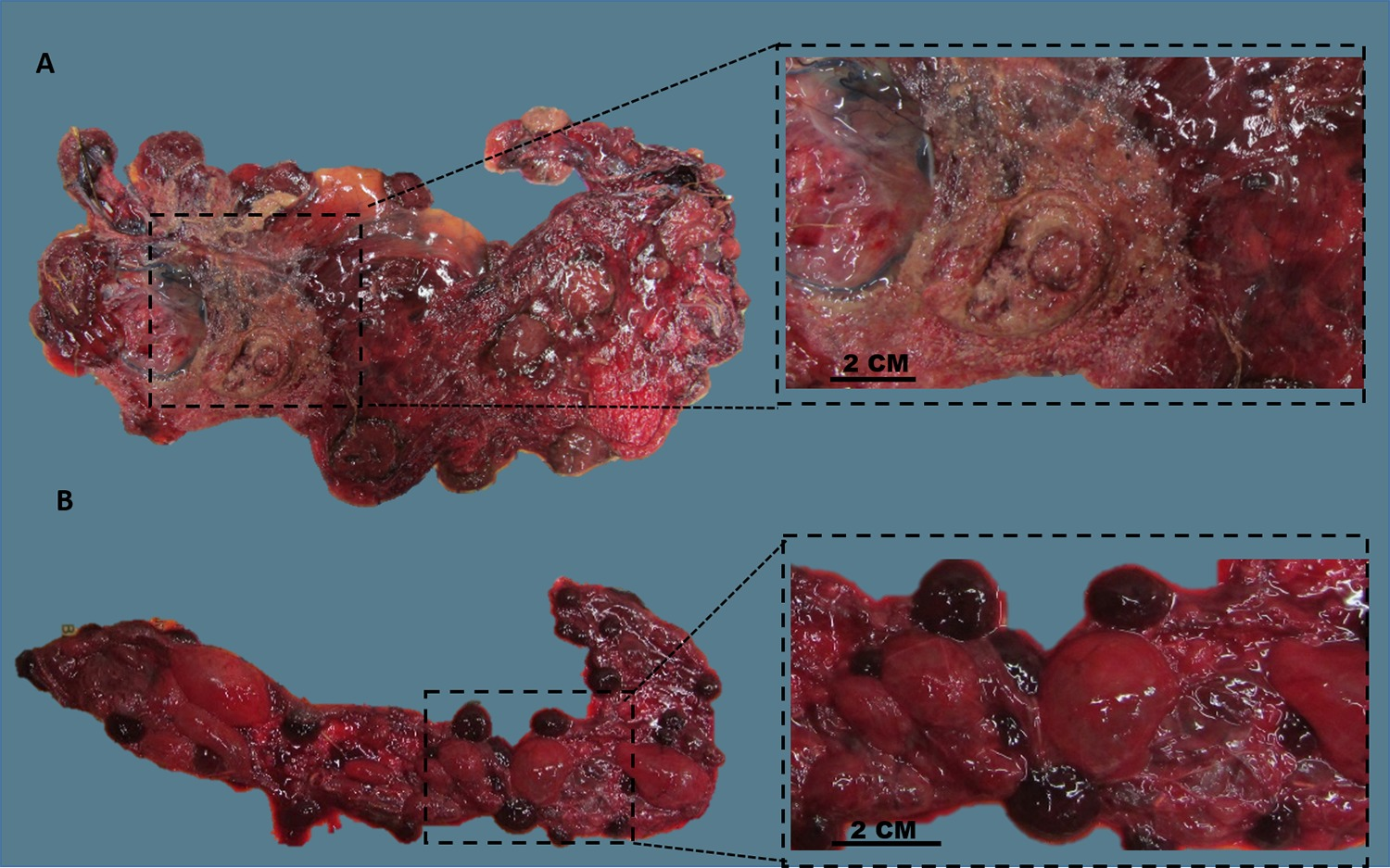

Supplement: S3 Fig — (A) Placenta wt-P2 showing the oedema and thickening of the whole placenta partially covered with a cream-colored exudate on the intercotyledonary area and dark red or grey cotyledons. (B) Placenta Neg-P2 showing red colour of the cotyledons, and thin and clear intercotyledonary membranes. (TIF) [file pone.0242526.s003.tif]

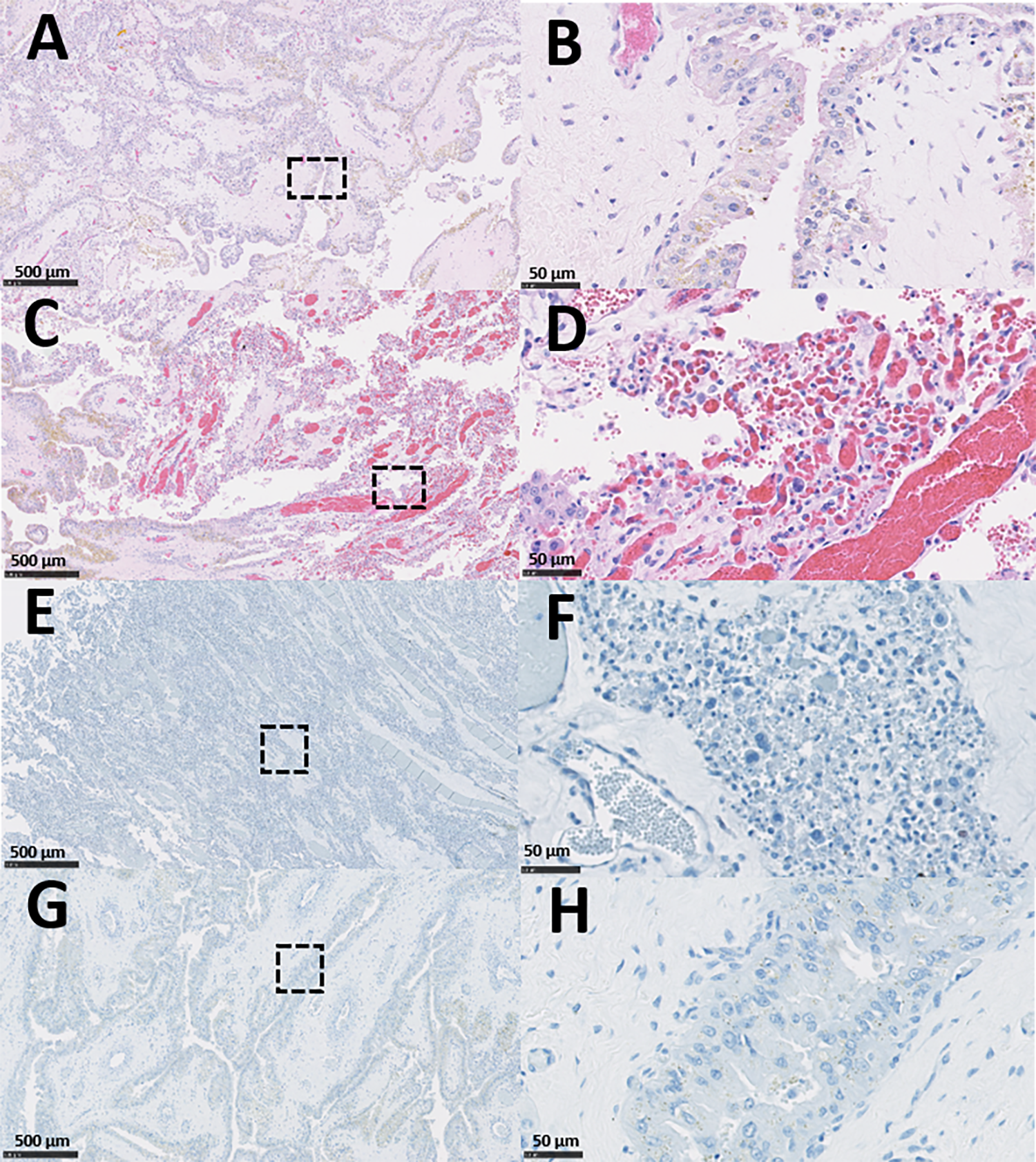

Supplement: S4 Fig — Ovine placentas infected with Neg-P1 (A, B, E and F) and Neg-P2 (C, D, G and H) strained with HE (A-D) and with IHC using genus-specific anti-LPS mAb 13/4 and counterstained with haematoxylin (E-H). (AB) Placenta Neg-P1: showing (A) placental epithelium showing typical trophoblastic cells. (C-D) Placenta Neg-P2: showing congestion of the blood vessel with absence of thrombosis and perivascular infiltration. (E-F) Placenta Neg-P1: showing (E) Negative immunolabelling of the trophoblast cells. (G-H) Placenta Neg-P2: Negative labelling of the trophoblast layer. The outlined black squares in the images on the left indicate the magnified area shown in the images on their immediate right. IHC anti-chlamydia mAb 13/4. (Scale bar. A, C, E and G: 500μm; B, D, F, and H: 50μm). (TIF) [file pone.0242526.s004.tif]

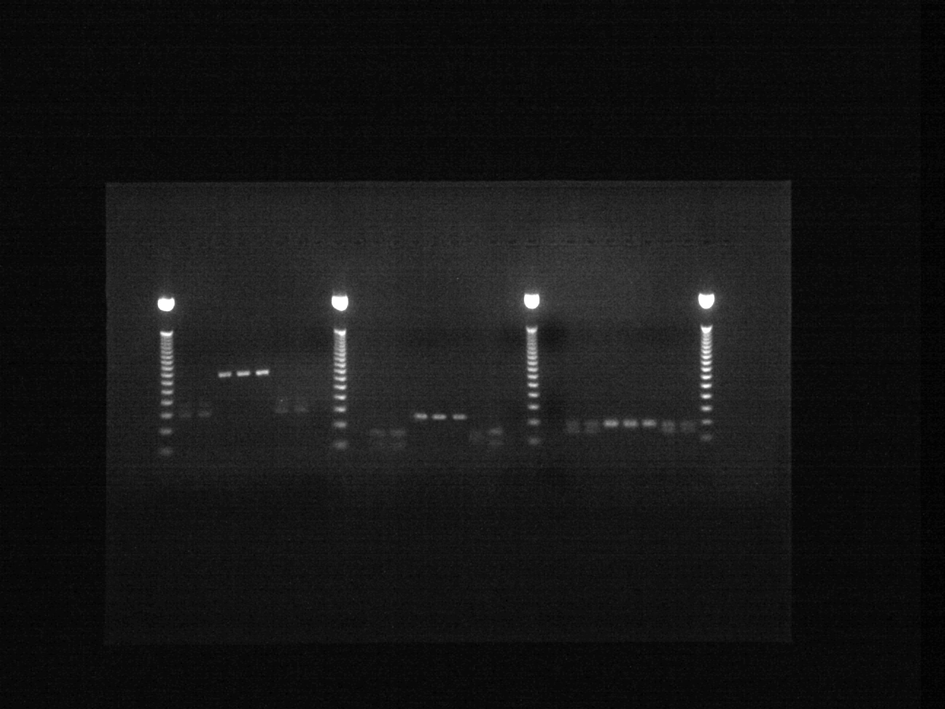

Supplement: S5 Fig — (TIF) [file pone.0242526.s005.tif]
