# Supplementary material for: The 1B vaccine strain of Chlamydia abortus produces placental pathology indistinguishable from a wild type infection
Source: PLoS One. 2020 Nov 16;15(11):e0242526. doi: 10.1371/journal.pone.0242526 (PMC7668586; doi:10.1371/journal.pone.0242526)
Supplement: Attachment — Submitted filename: PONE-D-20-27056_edited.pdf [file pone.0242526.s006.pdf]

PLOS ONE

The 1B vaccine strain of <i>Chlamydia abortus</i> produces placental pathology indistinguishable from a wild type infection

--Manuscript Draft--

Manuscript Number:	PONE-D-20-27056
Article Type:	Research Article
Full Title:	The 1B vaccine strain of <i>Chlamydia abortus</i> produces placental pathology indistinguishable from a wild type infection
Short Title:	Chlamydia abortus 1B vaccine strain placental pathology
Corresponding Author:	Gaston Caspe<br>Moredun Research Institute<br>Penicuik, UNITED KINGDOM
Keywords:	Chlamydia abortus; 1B vaccine strain; pathology; placenta; PCR-RFLP; histology; immunohistochemistry
Abstract:	Chlamydia abortus is one of the most commonly diagnosed causes of infectious abortion in small ruminants worldwide. Control of the disease (Enzootic Abortion of Ewes or EAE) is achieved using the commercial live, attenuated C. abortus 1B vaccine strain, which can be distinguished from virulent wild-type (wt) strains by polymerase chain reaction-restriction fragment length polymorphism (PCR-RFLP) analysis. Application of this typing method and whole-genome sequence analyses to cases of EAE in vaccinated and non-vaccinated animals have provided strong evidence that the 1B strain is not attenuated and can infect the placenta causing disease in some ewes. Therefore, the objective of this study was to characterise the lesions found in the placentas of ewes vaccinated with the 1B strain and to compare these to those resulting from a wt infection. A C. abortus -free flock of multiparous adult ewes was vaccinated twice, over three breeding seasons, each before mating, with the commercial C. abortus 1B vaccine strain (Cevac® Chlamydia, Ceva Animal Health Ltd.). In the second lambing season following vaccination, placentas (n=117) were collected at parturition and analysed by C. abortus -specific real-time quantitative PCR (qPCR). Two placentas, from a single ewe, which gave birth to live twin lambs, were found to be positive by qPCR and viable organisms were recovered and identified as vaccine type (vt) by PCR-RFLP, with no evidence of any wt strain being present. All cotyledons from the vt-infected placentas were analysed by histopathology and immunohistochemistry and compared to those from wt-infected placentas. Both vt-infected placentas showed lesions typical of those found in a wt infection in terms of their severity, distribution, and associated intensity of antigen labelling. These results conclusively demonstrate that the 1B strain can infect the placenta, producing typical EAE placental lesions that are indistinguishable from those found in wt infected animals.
Order of Authors:	Gaston Caspe
	Morag Livingstone
	David Frew
	Kevin Aitchison
	Sean Wattegedera
	Gary Entrican
	Javier Palarea-Albaladejo
	Tom McNeilly
	Elspeth Milne
	Neil Sargison
	Francesca Chianini

Powered by Editorial Manager® and ProduXion Manager® from Aries Systems Corporation

	David Longbottom
**Additional Information:**	
**Question**	**Response**
**Financial Disclosure**<br><br>Enter a financial disclosure statement that describes the sources of funding for the work included in this submission. Review the submission guidelines for detailed requirements. View published research articles from *PLOS ONE* for specific examples.<br><br>This statement is required for submission and **will appear in the published article** if the submission is accepted. Please make sure it is accurate.<br><br>**Unfunded studies**<br>Enter: *The author(s) received no specific funding for this work.*<br><br>**Funded studies**<br>Enter a statement with the following details:<br>• Initials of the authors who received each award<br>• Grant numbers awarded to each author<br>• The full name of each funder<br>• URL of each funder website<br>• Did the sponsors or funders play any role in the study design, data collection and analysis, decision to publish, or preparation of the manuscript?<br>• **NO** - Include this sentence at the end of your statement: *The funders had no role in study design, data collection and analysis, decision to publish, or preparation of the manuscript.*<br>• **YES** - Specify the role(s) played.<br><br>* typeset	ML, DF, KA, SRW, JPA, TNM, FC and DL are supported by the Scottish Government Rural and Environment Science and Analytical Services Division (RESAS) Strategic Research Programme, 2016–2021. SGC is supported by Intituto Nacional de Tecnologia Agropecuaria (INTA), Argentina. The funders had no role in study design, data collection and analysis, decision to publish, or preparation of the manuscript.
**Competing Interests**<br><br>Use the instructions below to enter a competing interest statement for this submission. On behalf of all authors, disclose any competing interests that could be perceived to bias this work—acknowledging all financial support and any other relevant financial or non-financial competing interests.	The authors have declared that no competing interests exist.

This statement **will appear in the published article** if the submission is accepted. Please make sure it is accurate. View published research articles from *PLOS ONE* for specific examples.<br><br>**NO authors have competing interests**<br><br>Enter: *The authors have declared that no competing interests exist.*<br><br>**Authors with competing interests**<br><br>Enter competing interest details beginning with this statement:<br><br>*I have read the journal's policy and the authors of this manuscript have the following competing interests: [insert competing interests here]*<br><br>* typeset	
**Ethics Statement**<br><br>Enter an ethics statement for this submission. This statement is required if the study involved:<br><br>• Human participants<br>• Human specimens or tissue<br>• Vertebrate animals or cephalopods<br>• Vertebrate embryos or tissues<br>• Field research<br><br>Write "N/A" if the submission does not require an ethics statement.<br><br>General guidance is provided below. Consult the submission guidelines for detailed instructions. **Make sure that all information entered here is included in the Methods section of the manuscript.**	Placentas used in the present study to test the presence of C. abortus 1B strain were collected from a flock of Texel-cross ewes that had been vaccinated with the commercial C. abortus 1B live, attenuated vaccine (see next section) on a working farm at parturition. These ewes had previously been purchased at a market from a Premium Health Scheme-accredited C. abortus-free flock. As no experimental procedures, sampling or any other interventions were conducted on these animals, and placentas only were collected as part of normal lambing, this part of the study was not subject to any Home Office licensing requirements.<br>Positive control formalin-fixed placental samples (wt-P1 and -P2) were used in this study for comparative purposes. These samples originated from a previously conducted study, from two ewes that had been experimentally inoculated with C. abortus strain S26/3 and both of which had aborted, one at 130 and the other at 133 days of gestation [15]. Negative control placental samples (Neg-P1 and -P2) also originating from this same study were taken from EAE-negative ewes. The study described here was carried out in strict accordance with the Animals (Scientific Procedures) Act 1986 and in compliance with all UK Home Office Inspectorate regulations. The experimental protocol was approved by the Moredun Experiments and Ethical Review Committee (Permit number: E30/17). All animals were monitored throughout the study for any clinical signs of ill health at least three times daily and any findings were recorded. Any animal found to be suffering or requiring treatment, for example from secondary bacterial infections, was given appropriate veterinary care (including use of antibiotics by a registered veterinary practitioner) in accordance with standard veterinary practice.

Powered by Editorial Manager® and ProduXion Manager® from Aries Systems Corporation

**Format for specific study types**<br><br>**Human Subject Research (involving human participants and/or tissue)**<br>• Give the name of the institutional review board or ethics committee that approved the study<br>• Include the approval number and/or a statement indicating approval of this research<br>• Indicate the form of consent obtained (written/oral) or the reason that consent was not obtained (e.g. the data were analyzed anonymously)<br><br>**Animal Research (involving vertebrate animals, embryos or tissues)**<br>• Provide the name of the Institutional Animal Care and Use Committee (IACUC) or other relevant ethics board that reviewed the study protocol, and indicate whether they approved this research or granted a formal waiver of ethical approval<br>• Include an approval number if one was obtained<br>• If the study involved *non-human primates*, add *additional details* about animal welfare and steps taken to ameliorate suffering<br>• If anesthesia, euthanasia, or any kind of animal sacrifice is part of the study, include briefly which substances and/or methods were applied<br><br>**Field Research**<br><br>Include the following details if this study involves the collection of plant, animal, or other materials from a natural setting:<br>• Field permit number<br>• Name of the institution or relevant body that granted permission	
**Data Availability**<br><br>Authors are required to make all data underlying the findings described fully available, without restriction, and from the time of publication. PLOS allows rare exceptions to address legal and ethical concerns. See the PLOS Data Policy and FAQ for detailed information.	Yes - all data are fully available without restriction

Powered by Editorial Manager® and ProduXion Manager® from Aries Systems Corporation

A Data Availability Statement describing where the data can be found is required at submission. Your answers to this question constitute the Data Availability Statement and **will be published in the article**, if accepted.<br><br>**Important:** Stating 'data available on request from the author' is not sufficient. If your data are only available upon request, select 'No' for the first question and explain your exceptional situation in the text box.<br><br>Do the authors confirm that all data underlying the findings described in their manuscript are fully available without restriction?	
**Describe where the data may be found in full sentences. If you are copying our sample text, replace any instances of XXX with the appropriate details.**<br><br>• If the data are **held or will be held in a public repository**, include URLs, accession numbers or DOIs. If this information will only be available after acceptance, indicate this by ticking the box below. For example: *All XXX files are available from the XXX database (accession number(s) XXX, XXX.*).<br>• If the data are all contained **within the manuscript and/or Supporting Information files**, enter the following: *All relevant data are within the manuscript and its Supporting Information files.*<br>• If neither of these applies but you are able to provide **details of access elsewhere**, with or without limitations, please do so. For example:<br><br>*Data cannot be shared publicly because of [XXX]. Data are available from the XXX Institutional Data Access / Ethics Committee (contact via XXX) for researchers who meet the criteria for access to confidential data.*<br><br>*The data underlying the results presented in the study are available from (include the name of the third party*	The data underlying the results presented in the study are available from Gaston Caspe (gaston.caspe@moredun.ac.uk).

and contact information or URL).<br>• This text is appropriate if the data are owned by a third party and authors do not have permission to share the data.<br><br>* typeset	
Additional data availability information:	

Manuscript
Click here to access/download;Manuscript;Manuscript.docx

The 1B vaccine strain of *Chlamydia abortus* produces placental pathology indistinguishable from a wild type infection

Short title: *Chlamydia abortus* 1B vaccine strain placental pathology

Sergio Gaston Caspe[1,2,3*], Morag Livingstone[1], David Frew[1], Kevin Aitchison[1], Sean Ranjan Wattegedera[1], Gary Entrican[1], Javier Palarea-Albaladejo[4], Tom Nathan, McNeilly[1], Elspeth Milne[2], Neil Donald Sargison[2], Francesca Chianini[1], David Longbottom[1].

[1] Moredun Research Institute, Penicuik, Midlothian, United Kingdom.

[2] Royal (Dick) School of Veterinary Studies, University of Edinburgh, Edinburgh, United Kingdom.

[3] Estación Experimental Mercedes, Instituto Nacional de Tecnología Agropecuaria (INTA), Mercedes, Corrientes, Argentina

[4] Biomathematics and Statistics Scotland, Edinburgh, United Kingdom

* Corresponding author E-mail: gaston.caspe@moredun.ac.uk (SGC)

Abstract

Chlamydia abortus is one of the most commonly diagnosed causes of infectious abortion in small ruminants worldwide. Control of the disease (Enzootic Abortion of Ewes or EAE) is achieved using the commercial live, attenuated *C. abortus* 1B vaccine strain, which can be distinguished from virulent wild-type (wt) strains by polymerase chain reaction-restriction fragment length polymorphism (PCR-RFLP) analysis. Application of this typing method and whole-genome sequence analyses to cases of EAE in vaccinated and non-vaccinated animals have provided strong evidence that the 1B strain is not attenuated and can infect the placenta causing disease in some ewes. Therefore, the objective of this study was to characterise the lesions found in the placentas of ewes vaccinated with the 1B strain and to compare these to those resulting from a wt infection. A *C. abortus*-free flock of multiparous adult ewes was vaccinated twice, over three breeding seasons, each before mating, with the commercial *C. abortus* 1B vaccine strain (Cevac® Chlamydia, Ceva Animal Health Ltd.). In the second lambing season following vaccination, placentas (n=117) were collected at parturition and analysed by *C. abortus*-specific real-time quantitative PCR (qPCR). Two placentas, from a single ewe, which gave birth to live twin lambs, were found to be positive by qPCR and viable organisms were recovered and identified as vaccine type (vt) by PCR-RFLP, with no evidence of any wt strain being present. All cotyledons from the vt-infected placentas were analysed by histopathology and immunohistochemistry and compared to those from wt-infected placentas. Both vt-infected placentas showed lesions typical of those found in a wt infection in terms of their severity, distribution, and associated intensity of antigen labelling. These results conclusively demonstrate that the 1B strain can infect the placenta, producing typical EAE placental lesions that are indistinguishable from those found in wt infected animals.

Introduction

Chlamydia abortus, an obligate intracellular Gram-negative bacterium, is the etiological agent of an abortifacient disease variously known as Enzootic Abortion of Ewes (EAE), Ovine Enzootic Abortion (OEA), ovine chlamydiosis or chlamydial abortion [1]. EAE is one of the most commonly diagnosed causes of infectious abortion in small ruminants around the world, with the exception of Australia and New Zealand [2, 3]. In the UK, around 8.6% of flocks have been estimated to be affected, with an associated annual economic impact of upwards of £20M [4], while a field study has estimated the costs associated with chlamydial abortion storms to be in the region of £2,163 per 100 ewes [5].

The disease is characterised by late-term abortions (generally occurring in the last 2-3 weeks before expected lambing), stillbirths, or the delivery of weak lambs that fail to survive beyond 48 hrs [6, 7]. The most significant sources of infection for transmission of the disease are the products of abortion, in particular, the heavily infected placentas, vaginal fluids, and the coats of dead fetuses or live lambs born from infected ewes. Bacterial loads present in or on these products of abortion/lambing are extremely high, reaching greater than 10^7 chlamydial genomes per µl of extracted material [7]. These products can contaminate the bedding and pasture with *C .abortus*, facilitating the transmission of infectious organisms to naïve animals through inhalation and ingestion [6].

The severe mixed infiltration of inflammatory cells, interstitial oedema and necrosis of the chorionic epithelium (trophoblast) during chlamydial infection affect the efficiency of the exchange of oxygenated blood and nutrients at the maternal-fetal interface, as well as the placental production of progesterone, which is responsible for the maintenance of pregnancy [8]. In addition, the ensuing vascular damage and thrombosis can cause ischaemic necrosis

resulting from blockage of the oxygenated blood supply [9, 10]. It is likely that a combination of all these factors results in the premature delivery of a dead fetus or weak lambs.

Control of infection can be achieved using the live attenuated temperature-sensitive *C. abortus* strain known as 1B, which has been successfully used in vaccines in many European countries [3, 11]. This mutant strain was derived from the virulent field strain AB7 by chemical mutagenesis [12]. However, a specific polymerase chain reaction-restriction fragment length polymorphism (PCR-RFLP) typing assay that specifically differentiates the 1B vaccine strain from a wild-type (wt) strain has been developed and used to provide evidence suggesting that the 1B strain has a role in causing disease [11, 13, 14]. A recent analysis of the genome of the 1B vaccine strain and another temperature-sensitive mutant strain (*C. abortus* strain 1H) that had reverted to virulence, revealed that they are genetically identical, suggesting that there was no genetic basis for any attenuation in the 1B strain [3]. Furthermore, the 1B vaccine strain has very recently been reported to have been recovered from unvaccinated animals that had been in contact with vaccinated animals and which had aborted as a result of EAE, suggesting that the 1B vaccine strain can be naturally-transmitted [3, 11]. However, there have been no studies investigating placental pathology resulting from infection with the vaccine strain. Therefore, the aim of this study was to characterise the lesions produced in the placentas of ewes vaccinated with the *C. abortus* 1B vaccine strain and compare them with those observed during a ~~wild-type~~ infection.

Materials and methods

Ethics statement

Placentas used in the present study to test the presence of *C. abortus* 1B strain were collected from a flock of Texel-cross ewes that had been vaccinated with the commercial *C.*

abortus 1B live, attenuated vaccine (see next section) on a working farm at parturition. These ewes had previously been purchased at a market from a Premium Health Scheme-accredited *C. abortus*-free flock. As no experimental procedures, sampling or any other interventions were conducted on these animals, and placentas only were collected as part of normal lambing, this part of the study was not subject to any Home Office licensing requirements.

Positive control formalin-fixed placental samples (wt-P1 and -P2) were used in this study for comparative purposes. These samples originated from a previously conducted study, from two ewes that had been experimentally inoculated with *C. abortus* strain S26/3 and both of which had aborted, one at 130 and the other at 133 days of gestation [15]. Negative control placental samples (Neg-P1 and -P2) also originating from this same study were taken from EAE-negative ewes. The study described here was carried out in strict accordance with the Animals (Scientific Procedures) Act 1986 and in compliance with all UK Home Office Inspectorate regulations. The experimental protocol was approved by the Moredun Experiments and Ethical Review Committee (Permit number: E30/17). All animals were monitored throughout the study for any clinical signs of ill health at least three times daily and any findings were recorded. Any animal found to be suffering or requiring treatment, for example from ~~secondary~~ bacterial infections, was given appropriate veterinary care (including use of antibiotics by a registered veterinary practitioner) in accordance with standard veterinary practice.

Collection of placentas and initial bacteriological screening

Placentas (n=117) were collected from 75 Texel-cross ewes (from the same flock) at parturition. These ewes had been vaccinated on-farm as part of standard farming practice with a commercial 1B vaccine (Cevac® Chlamydia, Ceva Animal Health Ltd.) administered

via the sub-cutaneous route according to the manufacturer's instructions. The ewes had been vaccinated twice (n=16 vaccinated in 2015 and 2017; n=59 vaccinated in 2016 and 2017), each approximately five weeks prior to mating. There were no reports of abortion occurring in this flock and all ewes lambed 'apparently healthy' live lambs. Placentas were collected at lambing, and each placenta was bagged and labelled immediately following delivery and transported directly to Moredun Research Institute for analysis. All placentas were assessed macroscopically for lesions and the percentage of the surface affected was estimated as described previously [16, 17]. Smears from placentas showing lesions consistent with EAE were stained by modified Ziehl Neelsen (mZN) to confirm the presence of chlamydial organisms [18, 19]. Samples of cotyledons and surrounding intercotyledonary membrane were excised from all placentas for the recovery and isolation of live *C. abortus* organisms, and for molecular and pathological analyses, as detailed in the next sections, ensuring that any areas exhibiting gross pathology were sampled.

Bacteriological isolation

Isolation of *C. abortus* was performed in HEp-2 (Human Epithelial type 2) cells as follows. A sample of ~~homogenize~~d placental cotyledon was ground up in 4 ml sucrose–phosphate–glutamate transport medium (~~SPG~~; [20]) using a mortar and pestle and sterile sand then centrifuged at 100 x *g*. The supernatant was collected before consecutive passages through 0.8 and 0.45 µm filters (Sterile Acrodisc® Syringe Filters, Ann Arbor, USA). After filtration, the sample was inoculated onto HEp-2 cells grown on coverslips in Trac bottles (Thermo Scientific, Newport, UK) in Iscove's Modified Dulbecco's Medium (IMDM, Thermo Fisher Scientific, New York, USA), supplemented with 2% heat-inactivated foetal calf serum (Merck Life Science UK Limited, Gillingham, UK), Trac bottles were centrifuged

at 3000 × *g* at room temperature for 2 × 15 min and incubated at 37 °C in 5% CO_2 for 2 h before replenishing with fresh medium and then maintained under the same conditions. After 72 h, coverslips were fixed in methanol, stained using Giemsa 'Gurr' (Merck Ltd., Poole, UK), and examined for the presence of chlamydial inclusions by light microscopy.

Placenta sampling and sensitivity of detection methods

As it was possible for ~~a minimum of~~ only one cotyledon to be positive for *C. abortus* in a pool of cotyledons, it was necessary to determine the sensitivity of the *C. abortus* quantitative real-time PCR (qPCR) (described below) ~~for identifying this one positive cotyledon~~. This was achieved in a series of spiking experiments testing different proportions of ~~one~~ *C. abortus* positive cotyledon ($6x10^6$ *C. abortus* genome copies per µL of total extracted DNA) with an increasing numbers of negative cotyledons from *C. abortus*-free placentas by qPCR following extraction of DNA from the pooled tissue. Ratios of positive: negative cotyledons ranging from 1:1 to 1:15 were tested. Using this methodology, it was determined that a single positive cotyledon could be detected from a pool of up to 15 cotyledons with 95% confidence [21].

Hence, one half of fifteen selected cotyledons was excised from each of the collected placentas and pooled for molecular analyses (see below). The remaining halves of each cotyledon were left *in situ* and the placentas were fixed in 10% neutral buffered formalin for haematoxylin and eosin staining (HE) and immunohistochemical studies (IHC).

Each pool of half-cotyledons (n=15) from each of the 117 placentas was finely chopped using new sterile disposable scalpels and forceps for each placenta. The resulting mix homogenised for 30 sec in a stomacher bag (Stomacher 80® Biomaster, Seward Limited, West Sussex, UK) at medium speed (265 rpm). Each mix was aliquoted into sterile 2 ml

tubes and stored at -20°C for qPCR and PCR-RFLP analyses and possible future bacteriological isolation.

Quantitative real-time PCR and RFLP analyses

Total DNA was extracted from isolated organisms and representative 20 mg samples of pooled placental material (n=15 cotyledons) using a DNeasy Blood & Tissue kit (Qiagen Ltd., Crawley, UK), according to the manufacturer's instructions. DNA samples were eluted in 200 µl of supplied buffer AE and stored at -20°C for molecular analyses.

Quantitative real-time PCR was carried out on all extracted placental DNA samples using primers based on the outer membrane protein gene OmpA, as described previously [7]. Briefly, the PCR reaction consisted of 2X TaqMan® universal PCR master mix (Applied Biosystems, Warrington, UK), OmpA forward primer (5'-CGGCATTCAACCTCGTT-3') and reverse primer (5'-CCTTGAGTGATGCCTACATTGG-3'), fluorescent probe (TaqMan® probe, 5' FAM-GTTAAAGGATCCTCCATAGCAGCTGATCAG-TAMRA 3'), 1 µL of DNA, and sterile Nuclease-Free water (Promega Corporation, Madison, USA) up to a final volume of 25 µL per sample. The thermal cycling conditions were 50ºC for 2 minutes; 95ºC for 10 minutes; 45 cycles of 95ºC for 15 seconds; and finally, 60ºC for 1 minute. Amplification and detection were performed using a QuantStudio 5 real-time PCR System (Applied Biosystems, Foster City, USA), following the manufacturer's standard protocols. Each sample was tested in triplicate and quantified against a standard curve (established with 10-fold concentrations ranging from 10^7 to 10^1 genome copies of *C. abortus* strain S26/3 per reaction). Concentrations of placental DNA were determined using a NanoDrop ND-100 (NanoDrop Technologies, Wilmington, USA) spectrophotometer, as previously published [7].

All qPCR-positive samples were analysed further by PCR-RFLP to confirm if the samples were of a vaccine type (vt), wt or mixed infection origin. PCR-RFLP analysis differentiates vt and wt strains of *C. abortus* based on mutations in restriction enzyme sites in three genes (*Sfc*I in CAB153, *Hae*III in CAB636 and *Sau3*AI in CAB648 in *C. abortus* strain S26/3)[14, 22] that ~~ablate~~ cleavage of PCR fragments derived from vt strains. The PCR primers used are detailed in Table 1, and all assay conditions were as previously published [14]. In addition to using genomic DNA prepared from the *C. abortus* 1B vaccine strain as a control, we also used the wild-type parental vaccine strain AB7 and UK wild-type strain S26/3 for comparison.

Table 1. PCR-RFLP primers and fragment sizes for differentiation of *C. abortus* 1B vaccine strain from wild-type strains.

CDS	Forward primer (5'-3')	Reverse primer (5'-3')	Amplicon size (bp)	Restriction endonuclease site	Restriction fragment sizes of PCR products (bp)	
					1B strain	Wild-type strain
CAB153	ATTAAAAGTAAGTGGAAAGATTTTACAACCTTA	TGAGGTCATACTTCTCTGTTTTGATTTTAT	319	*Sfc*I	319	177+142
CAB636	AGTTTGTACTTTGATGAGAATTCCAATG	TGAGGTCATACTTCTCTGTTTTGATTTTAT	118	*Hae*III	118	74+44
CAB648	TCACAAAAGCGATGCCCATC	TCTAAATCTCCGCATTCGG	177	*Sau3*AI	88+89	67+21+89

Histopathological examination

All of the cotyledons from qPCR and PCR-RFLP positive placentas were fixed in 10% buffered formalin, trimmed, dehydrated through graded alcohol and xylene, and embedded in paraffin wax. Sections were cut at a thickness of 4µm, stained with HE and examined histologically.

The lesions observed in vt placentas were compared to those obtained from typical wt-EAE infected placentas. The latter were obtained from two ewes experimentally infected with *C. abortus* strain S26/3 (wt-placenta 1 (P1) cotyledons, n=33; and wt-P2 cotyledons, n= 38). Placentas from two EAE-free ewes that lambed normally served as negative controls (Neg-P1 cotyledons, n=40 and Neg-P2 cotyledons, n=36).

Immunohistochemistry

Formalin-fixed paraffin-wax embedded cotyledons were sectioned at 5 µm thickness, mounted on Superfrost® slides (Menzel-Gläser, Braunschweig, Germany) and labelled with a mouse monoclonal antibody (mAb) raised against the lipopolysaccharide (LPS) of *C. abortus* S26/3 strain (mAb clone 13/4, Santa Cruz Biotechnology, Heidelberg, Germany), as previously described [23]. Briefly, after dewaxing in xylene and rehydration in successive passages in alcohol and water, the slides were treated with 3% hydrogen peroxide (Merck Life Science UK Limited, Saint Louis, Missouri, USA) in methanol (v/v) to inactivate any endogenous peroxidase activity. After washing with tris-buffered saline (TBS), non-specific binding was blocked with 20% normal goat serum in TBS for 30 min. After overnight incubation with primary anti-chlamydial LPS mAb (diluted 1/500 in TBS), the slides were washed twice with TBS. Next, goat anti-mouse IgG conjugate (Envision™+ System HRP

labelled polymer, Dako, Ely, UK) 1/20 (v/v) was applied for 30 min, according to manufacturer's instructions. After washing with TBS, the slides were incubated with aminoethyl carbazole alcohol soluble chromogen (AEC, Vector Laboratories, Peterborough, UK) for 8 min before washing in tap water and counterstaining with haematoxylin and mounted with mounting solution (Histomount™, Thermo Fisher Scientific, Camarillo, CA, USA). Negative control sections were prepared for each sample substituting the primary mAb 13/4 with IgG1 isotype control from murine myeloma clone MOPC 21 (Merck Life Science UK Limited, Saint Louis, Missouri, USA) diluted 1/250 in TBS. A positive control section of placental tissue from a known ovine chlamydial abortion case was included in every run.

Slides were examined and scored, with grading based on intensity and extent of immunolabelling by immunohistochemistry in the trophoblast and mesenchyme (IHC) or in blood vessels (VIHC), from 0 to 5 (0, no labelling; 1, focal; 2, 2 to 4 foci; 3, multifocal; 4, locally extensive or multifocal to diffuse/intense; 5, diffuse/ very intense).

Results

Gross pathology, isolation and qPCR analysis

Two of the 117 placentas examined showed macroscopic lesions consistent with *C. abortus* infection (Fig 1 and S1 Fig). Both placentas (vt-P1 and -P2) were positive by qPCR ($5.4x10^6$ and $2.6x10^5$ genome copies per µL of total extracted DNA, respectively) and originated from the same ewe (ewe number 6093), which lambed normally producing two live lambs. Live organisms were successfully isolated from these two placentas and b[illegible] were confirmed as qPCR positive for *C. abortus*. There were no gross lesions evident on any of the remaining 115 placentas, all of which were negative by qPCR (median and interquartile range (IQR) values equal to 30.79 and 99.07 genome copies per µL of total

extracted DNA respectively; mean ± SD = 3.61 ± 1.4 in natural logarithm scale). These results are graphically summarized in S2 Fig.

Fig 1. Placentas infected with vaccine-type and wild-type *C. abortus* strains, and negative control.

(A) Placenta vt-P1: showing the oedema and thickening of the whole placenta covering and the typical dark red or grey-whitish colouration of the cotyledons and partially covered with a cream-coloured exudate on the intercotyledonary areas. (B) Placenta wt-P1: showing the oedema and thickening of the whole placenta partially covered with a cream-coloured exudate on the intercotyledonary area and dark red and grey cotyledons. (C) Placenta Neg-P1: showing red colour of the cotyledons, and thin and clear and translucent intercotyledonary membranes.

Approximately 60% of vt-P1 showed lesions consistent with *C. abortus* infection. These changes were characterised by dark red or grey-whitish colouration of the cotyledons with an overlying creamy friable exudate that extended to the adjacent intercotyledonary areas. The intercotyledonary regions showed a thickened, oedematous appearance that is characteristic of EAE (Fig 1A). No gross lesions were evident on vt-P2 at the time of assessment and it was only subsequently that we noted vt-P2 was also positive for *C. abortus* following mZN and PCR analysis. After formalin fixation, we observed that approximately 5-10% of the surface area showed differences in colouration of the cotyledons, being paler than the rest of the cotyledons, and the intercotyledonary tissue in this area was slightly thickened (S1 Fig). Both placentas were positive by mZN and by qPCR, while bacterial loads in vt-P1 and vt-P2 were found to be high (5.4 x 10^6 and 2.6 x10^5 genome copies per µL of total

extracted DNA, respectively). Gross lesions in wt-infected placentas, wt-P1 (Fig 1B) and wt-P2 (S3 Fig), affected approximately 60% of the placental surface, showing dark red cotyledons and thickened red intercotyledonary membranes, with a grey or whitish exudate adherent on the surface. These wt lesions were indistinguishable from those observed in the vt placentas.

PCR-RFLP analysis of qPCR-positive placental samples

PCR-RFLP analysis of the isolated organism and the two qPCR-positive placental samples (vt-P1 and -P2) revealed restriction enzyme digestion patterns identical to those obtained for the *C. abortus* 1B vaccine strain rather than those obtained for wt strain S26/3 or wt vaccine parent strain AB7 (Fig 2). Specifically, we observed no cleavage of the 319 bp CAB153 and 118 bp CAB636 PCR fragments with restriction enzymes *SfcI* and *HaeIII*, respectively, with genomic DNA from either of the placental samples originating from ewe 6093. This contrast with the control wt samples where cleavage occurred. Similarly, the 177 bp CAB648 (and equivalent genes) PCR products derived from the two ewe 6093 placental samples (vt-P1 and -P2) were only cleaved once with *Sau3AI*, producing fragments of 89 and 88 bp in size, as also observed for the 1B vaccine strain. In contrast, for the wt strains the 88 bp fragment was cleaved further producing fragments of 67 and 21 bp. There was no evidence of mixed vt and wt PCR products present in either of the placental samples from ewe 6093.

Fig 2. PCR-RFLP analysis of *C. abortus*-positive placental samples.

Restriction enzyme digestion patterns for vt-P1 (lanes 4, 11 and 18) and vt-P2 (lanes 5, 12 and 19), wt-P1 (6, 13 and 20) and wt-P2 (7, 14 and 21), control wt *C. abortus* strains S26/3

(1,8 and 15) and AB7 (2, 9 and 16) and control *C. abortus* 1B vaccine strain (3, 10 and 17) of PCR fragments (see Materials and methods and Table 1) with enzymes *Sfc*I (lanes 1-7), *Hae*III (lanes 8-14) and *Sau3*AI (lanes 15-21).

Placental Histopathology

The placentas from ewe 6093 (vt-P1 and -P2) that were *C. abortus* qPCR-positive and PCR-RFLP vt-positive exhibited pathology typical of that observed in EAE following a wt *C. abortus* infection. Placentas vt-P1 and vt-P2 showed lesions indistinguishable from both wt placentas (wt-P1 and -P2). In vt-P1, histological lesions were multifocal to diffuse, suppurative and necrotising ~~placentitis~~ (Fig 3A-C) in the majority of the cotyledons (43/48)~~,~~ associated with degenerative to necrotising vasculitis and occasional fibrinoid thrombosis (Fig 4A-C). The chorionic epithelium (trophoblast) was severely affected, with extensive areas of necrosis, commonly associated with a suppurative inflammatory infiltrate. In affected areas, the trophoblast displayed ~~a~~ severe disruption with abundant necrotic cellular debris, occasional foci of mineralisation, and mixed inflammatory infiltration, chiefly comprising polymorphonuclear neutrophils (PMN). Immediately under the necrotic areas of the trophoblast, the basement membrane and lamina propria showed a densely packed layer of mixed inflammatory cells, predominantly ~~of~~ PMN. In cotyledons, multifocal vasculitis was also present. These foci were characterized by a mixed inflammatory infiltration of mononuclear cells morphologically resembling macrophages and lymphocytes and a small number of PMN in the tunica media and tunica adventitia, associated with mural necrosis and occasional occlusive fibrinoid thrombosis. Luminal occlusions in arteries and arterioles associated with the thrombosis ~~showed a~~ variable ~~degree~~ of severity. These occlusions were

accompanied by areas of ischemic necrosis, presenting as extensive areas of fibrinoid material, which consisted of cellular debris, karyorrhectic cells and degenerated PMN (Fig 3B, 3C and 4B). In the chorioallantoic mesenchyme, severe oedema was observed, with a mixed inflammatory infiltration composed mainly by macrophages-like cells, lymphocytic cells, and a small number of PMN (Fig 3B). In this area, occasional necrotizing vasculitis was also observed, with fibrinoid mural necrosis, and mixed inflammatory infiltration and lytic cellular debris in the lamina media and adventitia. Fibrinoid thrombosis in arteries and arterioles was variable in ~~degrees~~ of luminal occlusion (Fig 4A-C).

Lesions in placenta vt-P2 were also indistinguishable from wt infected placentas, showing severe, multifocal, suppurative, necrotising, placentitis (Fig 3D-F) in most of the cotyledons (43/52) associated with necrotizing vasculitis and thrombosis (Fig 4D). In the trophoblast of the cotyledons, multifocal necrosis was observed mainly associated with a severe infiltration of PMN and fewer lymphocytic cells (Fig 3F). The chorioallantoic mesenchyme showed interstitial oedema with a mixed, multifocal, moderate inflammatory infiltration, composed principally of macrophage-like cells and scarce PMN and lymphocytic cells. In blood vessels, arterioles showed mixed cell infiltration of PMN and scarce lymphocytic cells, fibrinoid necrosis of the tunica media and with occasional thrombosis resulting in variable degrees of luminal occlusion (Fig 4D).

Fig 3. Histopathological changes in the placentas of sheep infected with *C. abortus* vaccine-type strains.

(A, C and E) Placenta vt-P1: showing (A) necrosuppurative placentitis and (C) inflammatory infiltration forming a compact layer consisting primarily of large numbers of leucocytes in the epithelium of the cotyledon (arrow) attached to superficial amorphous necrosuppurative

material; and (E) mixed infiltration, primarily of PMN, in different stages of degeneration, and abundant necrotic debris. (B, D and F) Placenta vt-P2: showing (B) necrosuppurative placentitis and (D) extensive compact layer of leucocytes in the trophoblast and basement membrane (arrow); and (F) a heavy and compact suppurative infiltration. Black outlined areas shown in top images indicates areas expanded in images located immediately below. (Scale bar: A: 5 mm; B: 10 mm; C: 500µm; D: 1 mm; E, F: 50µm).

Fig 4. Histopathological changes in the placental blood vessels of sheep infected with *C. abortus* vaccine-type and wild-type strains.

(A-C) Placenta vt-P1: showing (A) necrosuppurative vasculitis, with severe mixed inflammatory infiltration (black arrow) and partially occlusive thrombosis (*); (B) partially occlusive fibrinoid thrombosis in multiple vessels (*) with revascularisation, and ischemic necrosis of the trophoblast (N); (C) severe fibrinoid necrotising arteritis displaying a dense band of amorphous and intensely eosinophilic material (orange arrow); (D) Placenta vt-P2: showing severe occlusive thrombosis, with neovascularization, mineralization of the thrombi (*). (E) Placenta wt-P1: showing severe fibrinoid necrotising mural arteritis (orange arrow), multiple foci of thrombosis (*), severe perivascular inflammatory infiltration (black arrow) and ischemic necrosis of the trophoblast (N). (F) Placenta wt-P2, severe necrotising arteritis, showing mural degeneration (orange arrow), mixed infiltration (black arrow) and ischemic necrosis of the surrounding tissue (N). Haematoxylin and eosin. (Scale bar: A, B: 100µm; C, D, E: 500µm; F: 250µm,).

Histopathologically, the lesions in both wt infected placentas (wt-P1 and -P2) were similar to the ones observed in the vt-placentas. wt-placentas displayed multifocal to diffuse,

suppurative and necrotising placentitis (29/33 cotyledons in wt-P1, and 38/38 in wt-P2) (Fig 5A-F) with foci of mineralization (Fig 5D), thrombosis and degenerative to necrotizing vasculitis (Fig 4E and F). Extended areas of trophoblast exhibited abundant necrotic material and intense leucocyte infiltration, primarily PMN, forming a dense band of leucocytes in the basement membrane (Fig 5A, B, C, and D). Chorioallantois mesenchyme showed interstitial oedema, foci of haemorrhage and multifocal mixed inflammatory infiltrate ~~compost by~~ macrophage-like and lymphocytic cells, and PMN (Fig 5C-F). In these areas, affected arterioles and arteries showed marked perivascular mixed inflammatory infiltration, occlusive thrombosis, and fibrinoid mural necrosis affecting the tunica intima and media of the vessels (Fig 4E and F). As noted in the vt-placentas, the degree of the luminal occlusion~~s~~ of the blood vessels ~~were~~ variable and was associated with extensive areas of necrosis in the trophoblast (Fig 5C and D, and Fig 4E and F). No EAE-associated lesions were observed in any of the negative control placentas (Neg-P1 and Neg-P2) (S4-FigA-D).

Fig 5. Histopathological changes in the placentas of sheep infected with *C. abortus* wild-type strains.

(A, C and E) Placenta wt-P1: note (A) necrosuppurative placentitis, (C) extensive, severe, suppurative infiltration of the trophoblast, necrotic material (N) in the luminal face of the layer and multiple haemorrhages in the basal tissue. (E) wt-P1: suppurative infiltration and necrotic debris in the trophoblast; (B, D and F) Placenta wt-P2, (B) necrosuppurative placentitis (D) fibrinoid necrosis of the trophoblast (N) with foci of mineralization (orange arrow) and suppurative infiltration ~~in~~ forming a dense layer of leucocytes in the basement membrane (black arrow), (F) suppurative infiltration containing PMN in different degrees of degeneration and necrotic debris. Black outlined areas in images represents areas expanded in

images located in their immediate below (Scale bar: A: 10 mm; B: 5 mm; C, D: 500µm; E, F: 50µm).

Placental immunohistochemistry

Immunohistochemical labelling for *C. abortus* using the genus-specific anti-LPS mAb 13/4 in the vt infected placentas revealed intense positivity in most cotyledons. Only five (5/48*)* cotyledons in the vt-P1 and three (3/52) in the vt-P2 appeared negative. In both vt placentas, the strongest labelling was found mainly in chorionic trophoblast cells with a distribution that was principally multifocal to coalescing (Fig 6A and D). In the chorioallantoic mesenchyme, the positivity was like the trophoblast, but the distribution was predominantly focal. Similar labelling was observed in the wt-P1 and wt-P2 placentas and only one of the cotyledons from placenta wt-P1 was negative. Both wt placentas also showed strong positive mainly multifocal to coalescing labelling in the trophoblast (Fig 6E and G) and moderate to intense multifocal labelling in the mesenchyme. In all the placentas evaluated, vt and wt, blood vessels were negative in most of the tissues, except for 4 arterioles (one in vt-P2, one in ~~of~~ wt-P1 and two in wt-P2), that showed focal positive labelling in the tunica intima of the arterioles. No positive IHC was observed in the cotyledons from the negative control placentas (Neg-P1 and Neg-P2) (S4 Fig E and H).

Fig 6. Immunohistochemical detection of chlamydial antigen in ovine placentas.

~~Ovine placentas infected with vt (A-D) and wt (E-H) *C. abortus*~~ were labelled by IHC using genus-specific anti-LPS mAb 13/4 and counterstained with haematoxylin. (A and B) Placenta vt-P1: showing (A) diffuse positive labelling in the cotyledonary trophoblast, with (B) intense and abundant labelling of the trophoblasti~~c~~ cells. (C and D) Placenta vt-P2: showing (C)

positive labelling of the trophoblast, with (D) the intense multifocal to coalescent labelling of the trophoblast cells. (E and F) Placenta wt-P1: showing (E) intense multifocal to coalescent immunolabelling (F) with suppurative infiltration in the trophoblast. (G and H) Placenta wt-P2: diffuse moderate labelling of the trophoblast, showing (H) intense labelling of the individual mononuclear cell surrounding by abundant cellular debris. The outlined black squares in the images on the left indicate the magnified area shown in the images on their immediate right. IHC anti-chlamydia mAb 13/4. (Scale bar. A, C, E and G: 500µm; B, D, F, and H: 50µm).

Discussion

Chlamydia abortus vaccines based on the *C. abortus* temperature-sensitive mutant strain 1B strain are widely used in the control of EAE [11]. The strain was generated through the chemical mutagenesis of a virulent field strain (AB7) and is reported as having restricted growth above 35°C so that it will not grow in sheep, which have a body temperature of 39.1°C [3, 24]. This means, in theory, that the strain would stimulate protective immunity without causing disease. However, recent studies have provided genetic evidence suggesting that there is no attenuation of this strain [3], with experimental and field evidence that the 1B vaccine strain can cause abortion in vaccinated flocks, as well as evidence of transmission of infection to unvaccinated animals in contact with vaccinated animals [3, 11, 13, 14]. While, such studies have focused on the association between infection and abortion [11, 14], there have been no studies describing any associated placental pathology with the 1B vaccine strain.

In this study, we examined 117 placentas from a flock of sheep that had been vaccinated twice with the 1B vaccine strain over different breeding seasons, to look for

evidence of pathology associated with the presence of the vaccine strain. No abortions occurred in this flock, with all ewes reported as delivering ‘apparently normal healthy’ live lambs. However, we did not have access to the lambs to follow up their clinical status and determine whether they were truly ‘healthy’ and free of *C. abortus* infection. From the initial assessment of the placentas we identified one (vt-P1) that exhibited gross pathology ~~covering~~ around 60% of the placental surface and which was characteristic of the pathology typically observed in cases of EAE. The presence of chlamydial organisms was confirmed in this case by mZN staining of a placental smear. ~~However,~~ smears of all of the other collected placentas revealed another placenta (vt-P2) to be also positive. As all sampling was undertaken blind it was only later that we determined that this additional placenta was from the same ewe. It is not unusual for the extent of gross pathology to vary in placentas from the same ewe and in different ewes that abort all lambs, or deliver a combination of dead, weak or ‘apparently normal’ lambs [1]. Generally, the more gross pathological damage that is observed (typically 60-100% of the placental surface) the more likely that an abortion will occur (due to placental insufficiency resulting in a lack of nutrients and oxygenated blood getting to the fetus), but conversely there are many instances where a heavily infected placenta is delivered following the birth of an ‘apparently normal’ live lamb. Indeed, in this study, although vt-P1 exhibited around 60% gross pathology, the ewe delivered what appeared to be normal healthy lambs. Thus, even though the animal delivered these healthy lambs the fact that the placenta was heavily infected and ~~live organisms~~ were recoverable and confirmed as vt, this represents an important source and risk for transmission of infection to naïve animals. Indeed, this risk could be considered greater than following an abortion event as it is possible that the farmer would not notice such a heavily infected placenta when the lamb was born alive and therefore ~~the placenta would possibly not be evaluated or a part of it sent~~ off to the local veterinary laboratory for diagnosis. Furthermore, a placenta that is less infected from a live lambing

would be even less likely to be noticed, such as vt-P2 in this study (5-10% coverage), and therefore also pose an important and significant risk factor for infection transmission. Thus, although the extent of visible gross pathology may be indicative of pregnancy outcome, this is not always the case, while delivery of live lambs alone is not a good indication that a flock is free of infection. Instead, if there is any hint of a possible chlamydial infection in a flock, a full histological examination of many of the placental cotyledons would ideally be required to properly determine the extent of infection and tissue damage. But of course, this is not routinely feasible in a diagnostic laboratory setting.

~~This~~ ~~tissue~~ damage, known as necrosuppurative placentitis, is caused by a combination of the action of the bacteria as well as the ensuing immunoreactivity associated with placental infection [16, 25, 26]. In the present study, even though the placenta infected with the vt strain showed lesions and was positive by PCR, mZN and IHC for *C. abortus*, the ewe gave birth to two 'normal' lambs. Both vt placentas presented different degrees of lesions, vt-P1 being more affected (60%) than the vt-P2 (5-10%); however, ~~these~~ were typical EAE lesions indistinguishable from those resulting from the wt S26/3 strain. This suggests that, at least in terms of gross placental pathology, the lesions found in these vt infected placentas are very similar to those resulting from a typical wt infection. Furthermore, although there is a difference in the ~~percentages~~ of the placenta affected, a very similar number of ~~organisms~~ was found by real-time qPCR within a specific pathological lesion. Considering that 15 cotyledons were randomly selected from each placenta, this may indicate that placental bacterial load is not ~~directly~~ related to the extent of the gross pathology or the clinical outcome, but is more a reflection of sampling, as suggested previously [6].

Quantitative PCR analysis of all of the placentas, ~~having determined the level of sensitivity for detection,~~ confirmed that only two placentas were positive for *Chlamydia abortus* (vt-P1 and vt-P2), while PCR-RFLP analysis confirmed the genotype in both cases to

be vt rather than wt. There was no evidence of any mixed infection in either of the samples, as observed in other studies [11, 13, 14, 22], suggesting that any observed gross pathology was due to the vaccine rather than a wild-type *C. abortus* strain. Furthermore, bacterial load estimated by qPCR also showed the presence of antigen in cotyledons to be very similar to those observed in a normal field infection resulting from a wt strain. While there were no abortions in this flock, possibly as a result of the two vaccinations giving added protective immunity, the identification of even a single animal with infection and live organisms in the placentas could be enough to cause transmission to other naïve animals, causing infection and possible abortion in the subsequent breeding season [3]. The fact that the vt strain identified in these placentas originated from the administered vaccine strain would then imply a possible risk of transmission of this vaccine strain to other unvaccinated naïve animals, as has been recently reported to occur in flocks in France [11].

Histologically, EAE typically shows a multifocal to diffuse, suppurative, necrotising placentitis resulting from infection of the placental chorionic epithelium and caused by a combination of the action of the bacteria as well as the ensuing immunoreactive response. Infection is thought to occur following the transmission of chlamydial EBs from the uterine caruncle to the trophoblast cells of the chorionic epithelium in the fetal cotyledon, probably as a result of the leakage of blood from capillaries in the maternal caruncle septal tips that occurs around day 60 of gestation [10]. From here the chlamydial EBs replicate and form the readily visible cytoplasmic inclusions observed in both the wt and vt placentas in this study, as well as in other published studies [16]. These pathological changes have generally been reported as occurring after around day 84-90 of gestation [10]. As the surrounding trophoblast is destroyed by the invading chlamydial organisms the inclusions become less defined, with a more diffuse staining resulting from release of the EBs being mostly evident. During this time infection spreads to the peri-placentome and then to the intercotyledonary

regions, resulting in epithelial damage, oedema and inflammation that gives rise to the characteristic reddened and thickened placental membranes that are associated with this disease [6, 9, 16, 17] and which was also observed in this study. Histological assessment of the two vt positive placentas revealed a mixed inflammatory cell infiltrate, characterised mainly by PMN and a lesser number of macrophages and lymphocytic cells, with vasculitis and occasional occlusive fibrinous thrombosis. Furthermore, the lesions from these two placentas were found to be indistinguishable from each other, differing only in terms of the extent of tissue damage, as well as indistinguishable from those observed in classical wt disease, either as judged by the positive control samples used in the present study (wt-P1 and wt-P2) or as reported previously in other studies [10,15]. Such observed differences in placental pathology in multiple placentas from the same animal is a common observation for this disease [6, 17]. Thus, overall, the results suggest that there is no difference between the lesions resulting from a normal wild-type infection and those resulting from an infection caused by administration of the commercial *C. abortus* 1B vaccine strain.

Conclusion

This study has provided strong molecular evidence for the presence of ~~only~~ the *C. abortus* live attenuated 1B vaccine strain in the placentas of a ewe exhibiting gross pathology characteristic of that observed following a wt infection. Furthermore, the bacterial load was similar to that observed in ~~such~~ typical wt cases. Histological and immunohistochemical analysis showed no discernible difference in pathology from that observed in classical cases of EAE. Therefore, the results suggest that the vt strain can cause the typical placental lesions that are observed following a wt strain infection in ewes. These results complement those of an earlier study [17] where administration of a high dose of a virulent wt strain (same wt

strain as used in this study) stimulated protective immunity resulting in a much-reduced abortion level. Combined with evidence that the live vaccine strain is not attenuated [3], this suggests that the vaccine strain provides protection through the high doses that are administered rather than because of any attenuation. Although there was no abortion associated with the pathology in this study, the fact that a high bacterial load was present in the placentas has potentially important implications regarding transmission of the live vaccine to other unvaccinated naïve animals, where it could result in disease and abortion. Similar consideration should be given in terms of human health as the pathogen is zoonotic.

Conflict of interest statement

None of the authors of this study have any financial or personal relationship with other people or organisations that could inappropriately influence or bias the content of this publication.

Acknowledgements

The authors would like to thank Val Forbes, Clare Underwood, Alice Plat and Moredun Bioservices for valuable technical assistance in helping with the processing of placentas.

References

1. Longbottom D, Coulter LJ. Animal chlamydioses and zoonotic implications. J Comp Pathol. 2003;128(4):217-44. doi: 10.1053/jcpa.2002.0629.

2. Rodolakis A, Laroucau K. Chlamydiaceae and chlamydial infections in sheep or goats. Vet Microbiol. 2015;181(1-2):107-18. doi: 10.1016/j.vetmic.2015.07.010.

3. Longbottom D, Sait M, Livingstone M, Laroucau K, Sachse K, Harris SR, et al. Genomic evidence that the live *Chlamydia abortus* vaccine strain 1B is not attenuated and has the potential to cause disease. Vaccine 2018;36(25):3593-3598. doi: 10.1016/j.vaccine.2018.05.042.

4. Wood R. Enzootic abortion costs home industry £20m pa. Farmer Weekly 1992; 117:60.

5. Robertson A, Handel I, Sargison ND. General evaluation of the economic impact of introduction of *Chlamydia abortus* to a Scottish sheep flock. Vet Rec Case Rep. 2018;6(3):e000689. doi:10.1136/vetreccr-2018-000689

6. Livingstone M, Wheelhouse N, Ensor H, Rocchi M, Maley S, Aitchison K, et al. Pathogenic outcome following experimental infection of sheep with *Chlamydia abortus* variant strains LLG and POS. PLoS One. 2017;12(5):e0177653. doi: 10.1371/journal.pone.0177653.

7. Livingstone M, Wheelhouse N, Maley SW, Longbottom D. Molecular detection of *Chlamydophila abortus* in post-abortion sheep at oestrus and subsequent lambing. Vet Microbiol. 2009;135(1-2):134-141. doi: 10.1016/j.vetmic.2008.09.033.

8. Essig A, Longbottom D. *Chlamydia abortus*: New Aspects of Infectious Abortion in Sheep and Potential Risk for Pregnant Women. Curr Clin Microbiol Rep. 2015;2(1):22-34. doi: 10.1007/s40588-015-0014-2.

9. Sammin D, Markey B, Bassett H, Buxton D. The ovine placenta and placentitis—A review. Vet Microbiol. 2009;135(1-2):90-97. doi: 10.1016/j.vetmic.2008.09.054.

10. Buxton D, Barlow RM, Finlayson J, Anderson IE, Mackellar A. Observations on the pathogenesis of *Chlamydia psittaci* infection of pregnant sheep. J Comp Pathol. 1990;102(2):222-237. doi: 10.1016/S0021-9975(08)80127-2.

11. Laroucau K, Aaziz R, Vorimore F, Menard MF, Longbottom D, Denis G. Abortion storm induced by the live *C. abortus* vaccine 1B strain in a vaccinated sheep flock, mimicking a natural wild-type infection. Vet Microbiol. 2018; 225:31-33. doi: 10.1016/j.vetmic.2018.09.012.

12. Rodolakis A, Souriau A. Response of ewes to temperature-sensitive mutants of *Chlamydia psittaci* (var *ovis*) obtained by NTG mutagenesis. Ann Rech Vet. 1983;14(2):155-161.

13. Sargison ND, Truyers IG, Howie FE, Thomson JR, Cox AL, Livingstone M, et al. Identification of the 1B vaccine strain of *Chlamydia abortus* in aborted placentas during the investigation of toxaemic and systemic disease in sheep. N Z Vet J. 2015;63(5):284-287. doi: 10.1080/00480169.2015.1018365.

14. Wheelhouse N, Aitchison K, Laroucau K, Thomson J, Longbottom D. Evidence of *Chlamydophila abortus* vaccine strain 1B as a possible cause of ovine enzootic abortion. Vaccine. 2010;28(35):5657-5663. doi: 10.1016/j.vaccine.2010.04.114.

15. Livingstone M, Aitchison K, Wattegedera S, Rocchi M, Wilson K, Sait M, et al. Development of a new vaccine to protect sheep from ovine enzootic abortion. In:

Proceeding of the Fifth European Meeting on Animal Chlamydioses. Odessa, Ukraine; 2018. pp. 25-26.

16. Buxton D, Anderson IE, Longbottom D, Livingstone M, Wattegedera S, Entrican G. Ovine chlamydial abortion: characterization of the inflammatory immune response in placental tissues. J Comp Pathol. 2002;127(2-3):133-141. doi: 10.1053/jcpa.2002.0573.

17. Longbottom D, Livingstone M, Maley S, van der Zon A, Rocchi M, Wilson K, et al. Intranasal infection with *Chlamydia abortus* induces dose-dependent latency and abortion in sheep. PLoS One. 2013;8(2):e57950. doi: 10.1371/journal.pone.0057950.

18. Sachse K, Vretou E, Livingstone M, Borel N, Pospischil A, Longbottom D. Recent developments in the laboratory diagnosis of chlamydial infections. Vet Microbiol. 2009;135(1-2):2-21. doi: 10.1016/j.vetmic.2008.09.040.

19. Stamp J, McEwen A, Watt J, Nisbet D. Enzootic abortion in ewes; transmission of the disease. Vet Rec. 1950;62(17):251-254.

20. Spencer WN, Johnson FWA. Simple transport medium for the isolation of *Chlamydia psittaci* from clinical material. Vet Rec. 1983;113: 535–536.

21. Sergeant E. Epitools epidemiological calculators [cited 27 August 2020]. In: AusVet Animal Health Services and Australian Biosecurity Cooperative Research Centre for Emerging Infectious Disease Available from: https://epitools.ausvet.com.au/.

22. Laroucau K, Vorimore F, Sachse K, Vretou E, Siarkou VI, Willems H, et al. Differential identification of *Chlamydophila abortus* live vaccine strain 1B and *C. abortus* field isolates by PCR-RFLP. Vaccine. 2010;28(35):5653-5656. doi: 10.1016/j.vaccine.2010.06.064.

23. Buxton D, Rae AG, Maley SW, Thomson KM, Livingstone M, Jones GE, et al. Pathogenesis of *Chlamydia psittaci* infection in sheep: detection of the organism in a serial study of the lymph node. J Comp Pathol. 1996;114(3):221-230. doi: 10.1016/s0021-9975(96)80044-2.

24. Reece WO, Erickson H, Goff J, Uemura E (Eds). Body temperature and its regulation. In: Dukes' physiology of domestic animals, 13th Ed. Oxford, England : Wiley Blackwell; 2015. pp. 149-154.

25. Entrican G, Wattegedera S, Wheelhouse N, Allan A, Rocchi M. Immunological paradigms and the pathogenesis of ovine chlamydial abortion. Am J Reprod Immunol. 2010;64(4):287-94. doi: 10.1111/j.1600-0897.2010.00907.x.

26. Wattegedera SR, Doull LE, Goncheva MI, Wheelhouse NM, Watson DM, Pearce J, et al. Immunological Homeostasis at the Ovine Placenta May Reflect the Degree of Maternal Fetal Interaction. Front Immunol. 2018;9:3025. doi: 10.3389/fimmu.2018.03025.

Supporting information

S1 Fig. Placentas vt-P2 from ewes infected with vt strain formalin-fixed.

Placenta vt-P2, note the difference in the coloration of the cotyledons and the thickening of the intercotyledonary membranes of the marked areas (black circles).

S2 Fig. Boxplot. Genome copies of *C. abortus* per µl of placental DNA.

Boxplot showing the number of genome copies (natural logarithmic scale) of *C. abortus* per µl of extracted genomic DNA in the 117 placentas analysed by qPCR. The two outlier points correspond to vt-P1 and vt-P2.

S3 Fig. Placentas from ewes infected with *C. abortus* s26/3 wild-type strains, and negative control.

(A) Placenta wt-P2 showing the oedema and thickening of the whole placenta partially covered with a cream-colored exudate on the intercotyledonary area and dark red or grey cotyledons. (B) Placenta Neg-P2 showing red colour of the cotyledons, and thin and clear intercotyledonary membranes.

S4 Fig. H[illegible]nd IHC in ovine placentas.

Ovine placentas infected with Neg-P1 (A, B, E and F) and Neg-P2 (C, D, G and H) strained with HE (A-D) and with IHC using genus-specific anti-LPS mAb 13/4 and counterstained with haematoxylin (E-H). (AB) Placenta Neg-P1: showing (A) placental epithelium showing typical trophoblastic cells. (C-D) Placenta Neg-P2: showing congestion of the blood vessel with absence of thrombosis and perivascular infiltration. (E-F) Placenta Neg-P1: showing (E)

Negative immunolabelling of the trophoblast cells. (G-H) Placenta Neg-P2: Negative labelling of the trophoblast. The outlined black squares in the images on the left indicate the magnified area shown in the images on their immediate right. IHC anti-chlamydia mAb 13/4. (Scale bar. A, C, E and G: 500µm; B, D, F, and H: 50µm).

S5-raw-gel-image.tif. PCR-RFLP analysis of *C. abortus*-positive placental samples. Original agarose gel visualised with uv transilluminator and image captured on a Syngene G:BOX F3. Restriction enzyme digestion patterns for vt-P1 (lanes 4, 11 and 18) and vt-P2 (lanes 5, 12 and 19), wt-P1 (6, 13 and 20) and wt-P2 (7, 14 and 21), control wt *C. abortus* strains S26/3 (1,8 and 15) and AB7 (2, 9 and 16) and control *C. abortus* 1B vaccine strain (3, 10 and 17) of PCR fragments with enzymes *Sfc*I (lanes 1-7), *Hae*III (lanes 8-14) and *Sau3*AI (lanes 15-21).

Figure 1

Click here to access/download;Figure;Fig1.tif

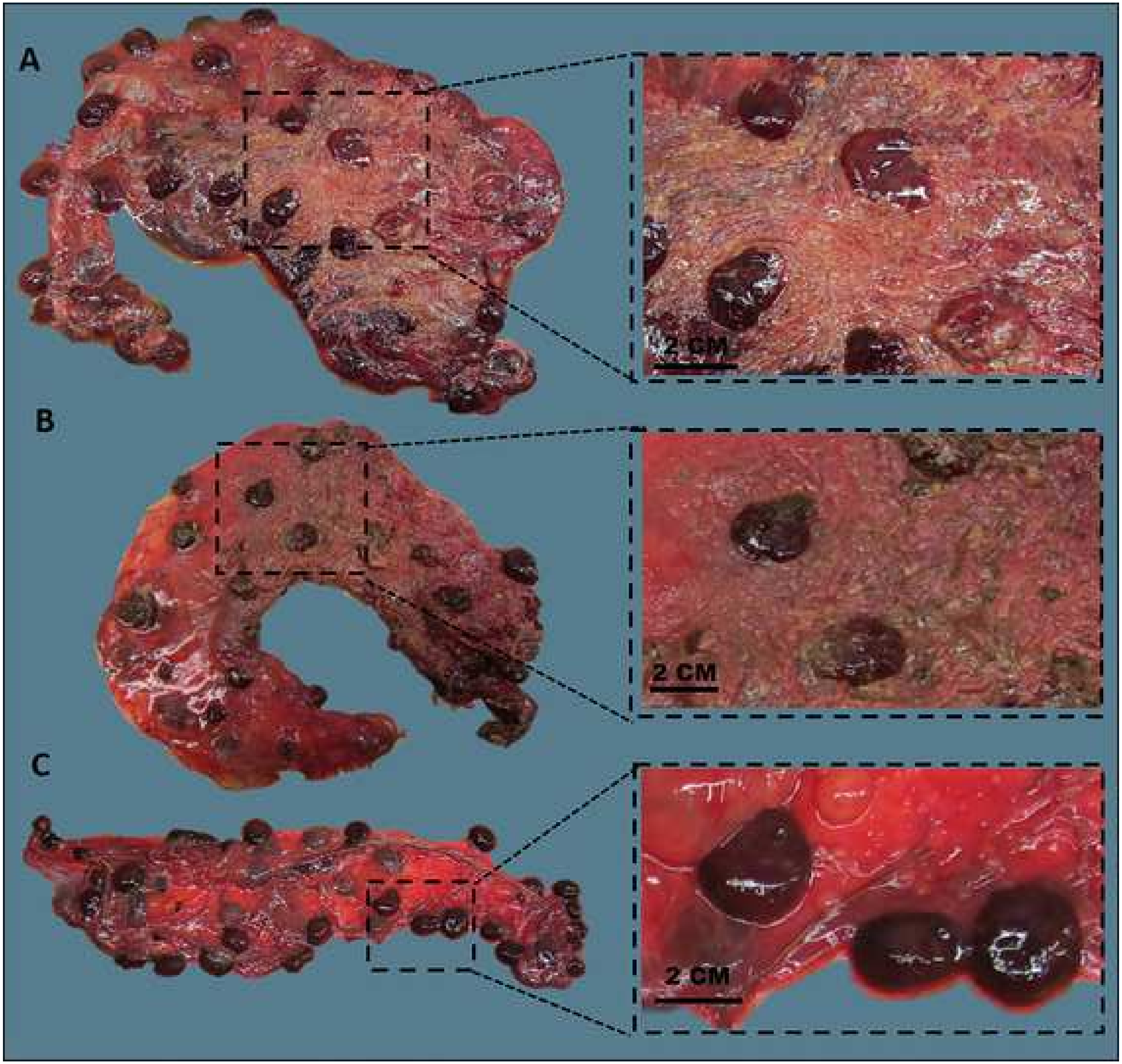

Figure 2

Click here to access/download;Figure;Fig2.tif

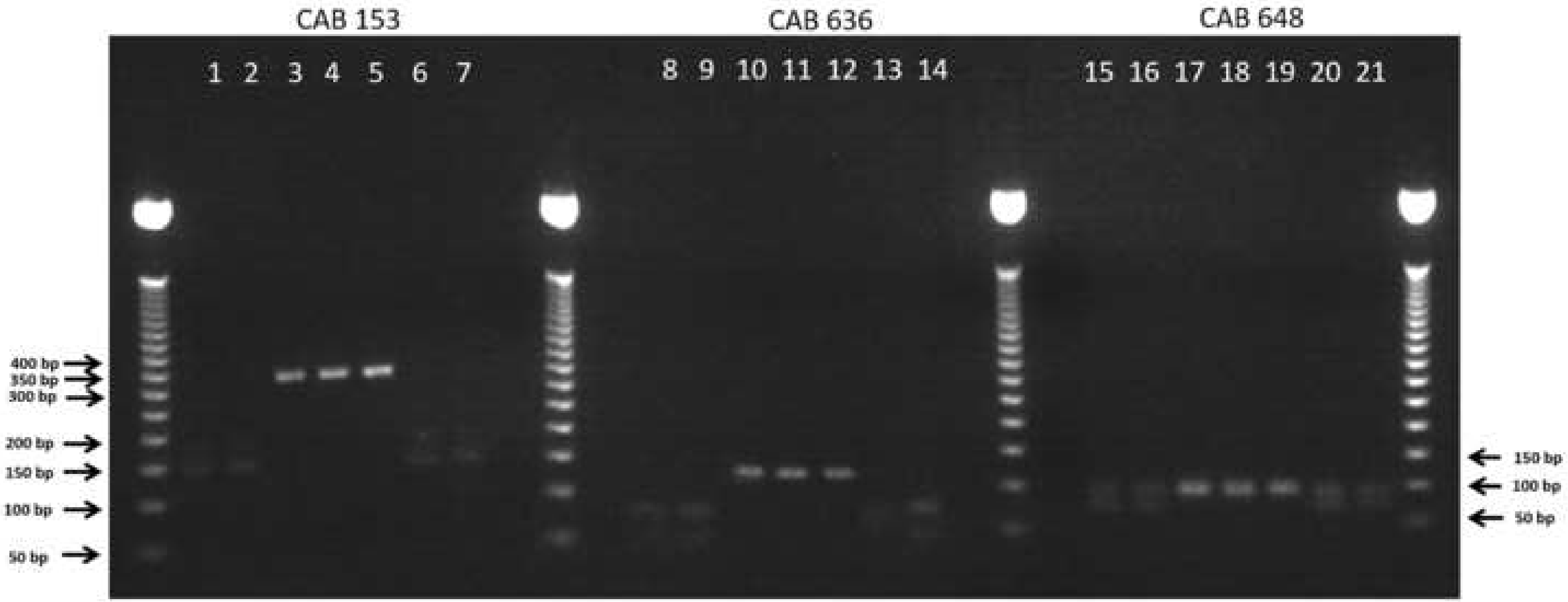

Figure 3

Click here to access/download;Figure;Fig3.tif

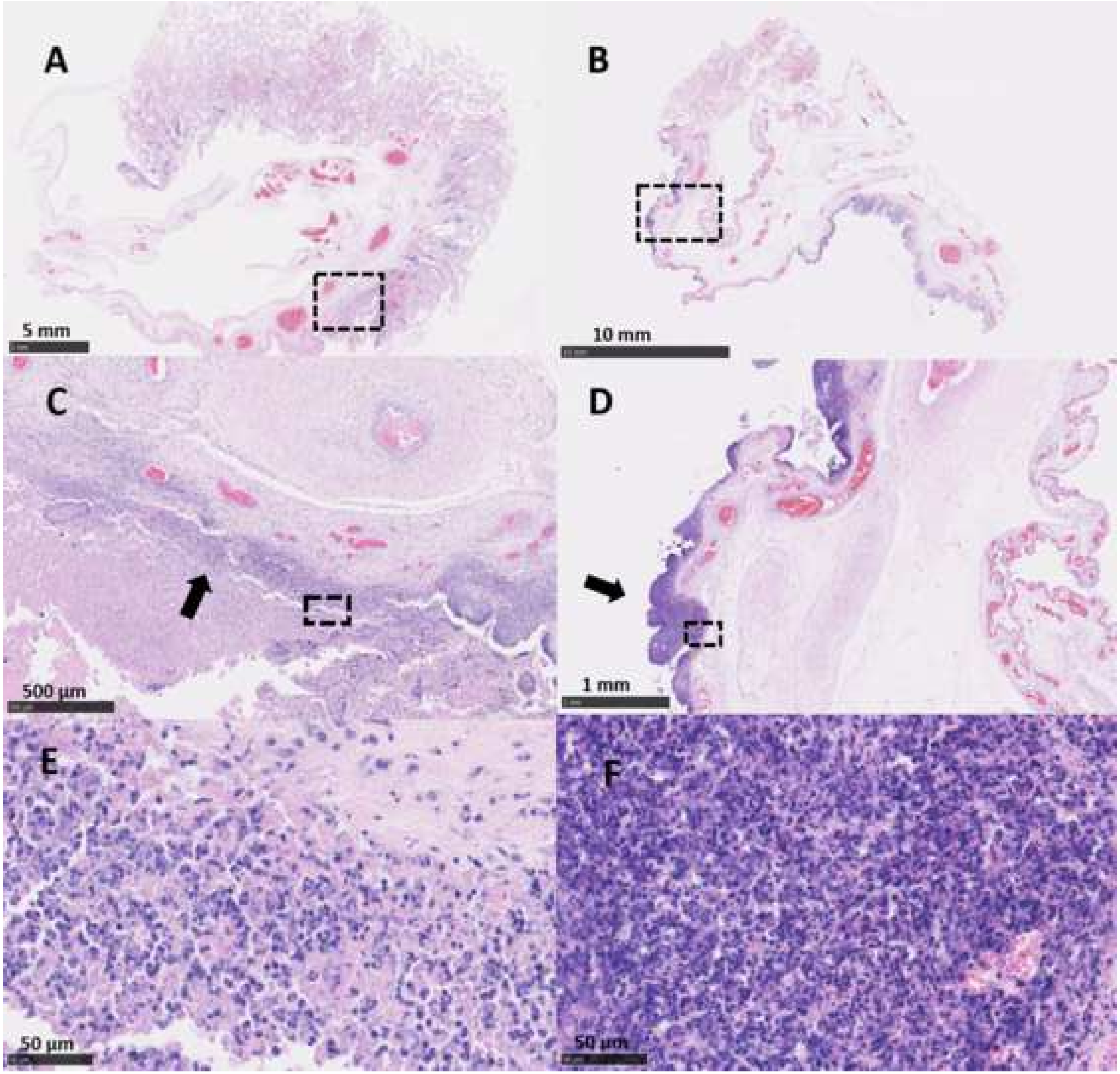

Figure 4

Click here to access/download;Figure;Fig4.tif

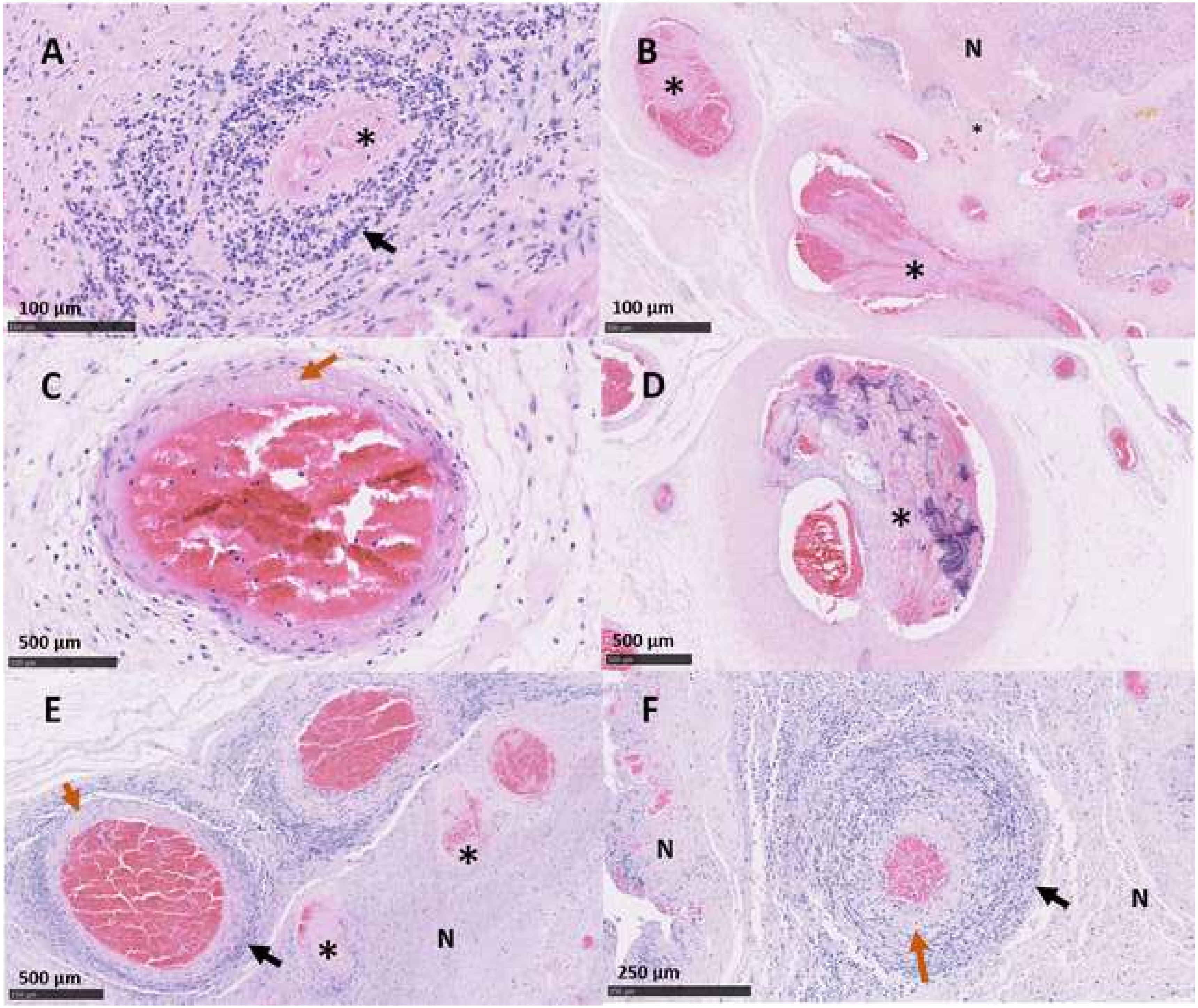

 Click here to access/download;Figure;Fig5.tif

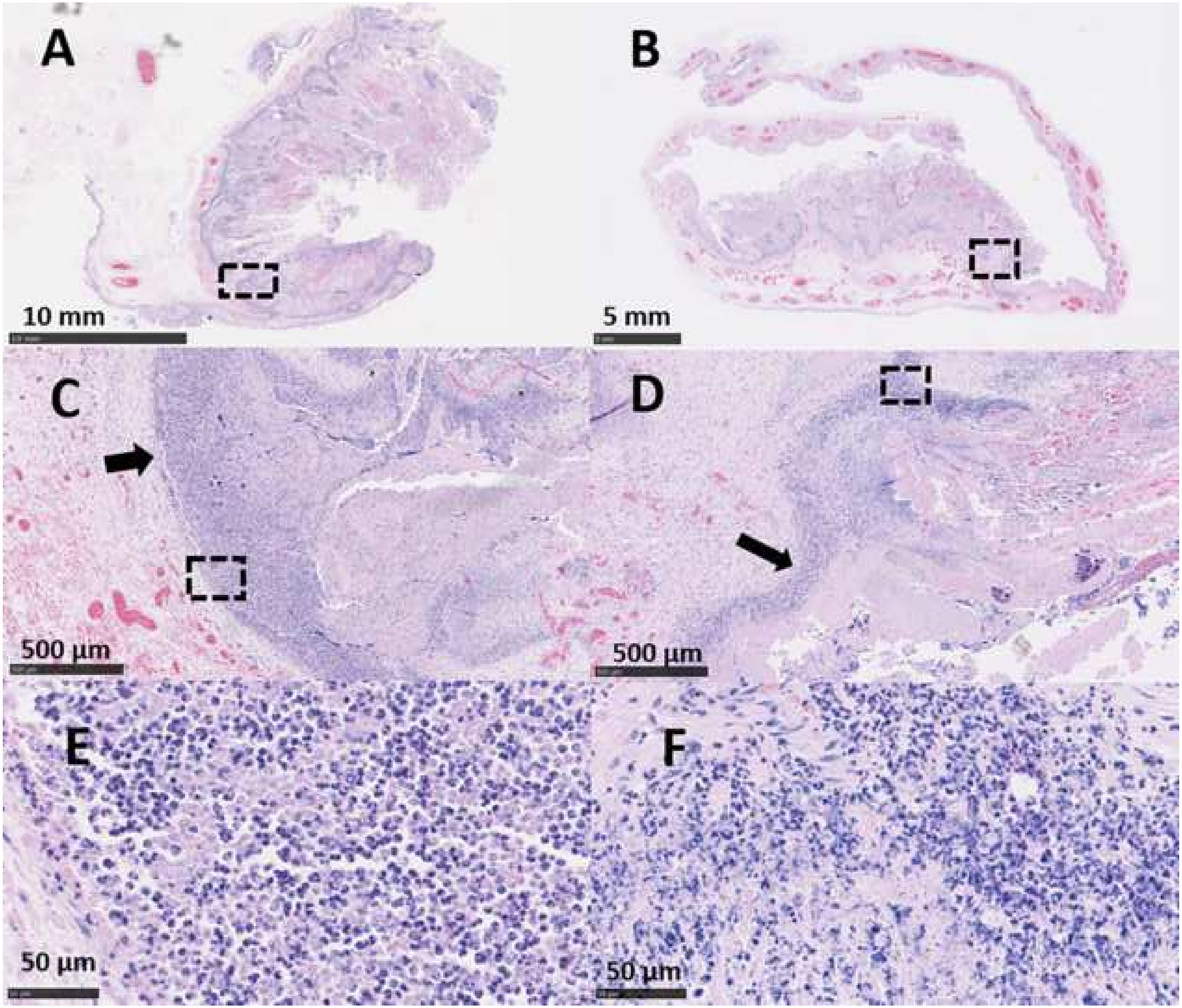

Figure 6

Click here to access/download;Figure;Fig6.tif

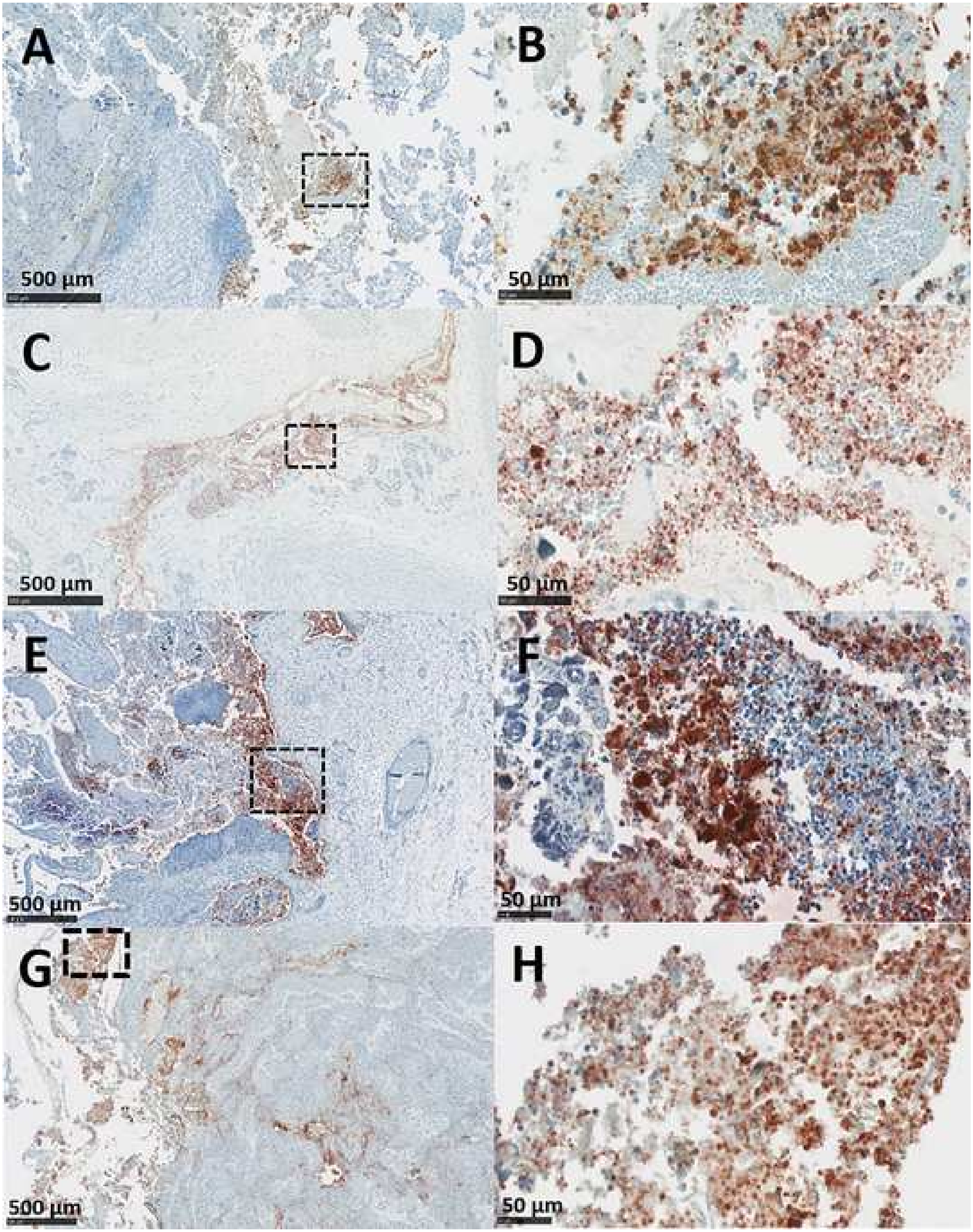

S1-Fig

Click here to access/download
Supporting Information
S1-Fig.tif

S2-Fig

Click here to access/download
Supporting Information
S2-Fig.tif

S3-Fig

Click here to access/download
Supporting Information
S3-Fig.tif

S4-Fig

Click here to access/download
Supporting Information
S4-Fig.tif

S5-raw-gel-image

Click here to access/download
Supporting Information
S5-raw-gel-image.tif
